# Recent Progress of Reclassification of the Genus *Streptomyces*

**DOI:** 10.3390/microorganisms11040831

**Published:** 2023-03-24

**Authors:** Hisayuki Komaki

**Affiliations:** Biological Resource Center, National Institute of Technology and Evaluation (NBRC), Chiba 292-0818, Japan; komaki-hisayuki@nite.go.jp

**Keywords:** classification, postgenome, rank elevation, subspecies, *Streptomyces*, synonym

## Abstract

The genus *Streptomyces* is a representative group of actinomycetes and one of the largest taxa in bacteria, including approximately 700 species with validly published names. Since the classification was mainly based on phenotypic characteristics in old days, many members needed to be reclassified according to recent molecular-based taxonomies. Recent developments of molecular-based analysis methods and availability of whole genome sequences of type strains enables researchers to reclassify these phylogenetically complex members on a large scale. This review introduces reclassifications of the genus *Streptomyces* reported in the past decade. Appropriately 34 *Streptomyces* species were transferred to the other genera, such as *Kitasatospora*, *Streptacidiphilus*, *Actinoalloteichus* and recently proposed new genera. As a result of reclassifications of 14 subspecies, the genus *Streptomyces* includes only four subspecies at present in practice. A total of 63 species were reclassified as later heterotypic synonyms of previously recognized species in 24 published reports. As strong relationships between species and the secondary metabolite-biosynthetic gene clusters become clarified, appropriate classifications of this genus will not only contribute to systematics, but also provide significant information when searching for useful bioactive substances.

## 1. Introduction

The genus *Streptomyces* is a representative group of actinomycetes. This genus is aerobic, Gram stain-positive, non-acid fast and multicellular bacteria that form extensively branched substrate and aerial mycelia with complex lifecycles. The aerial mycelium forms chains of three to many spores at maturity. Streptomycetes can be isolated in high numbers from soil, which is their primary natural habitat, and play an important role in the decomposition of organic matter such as leaf litters in soil, contributing to the fertility of soil. Although this genus includes plant pathogens and human pathogens, it is mainly characterized by the potential to produce a large variety of chemically different secondary metabolites, such as antibiotics.

**Figure 1:**
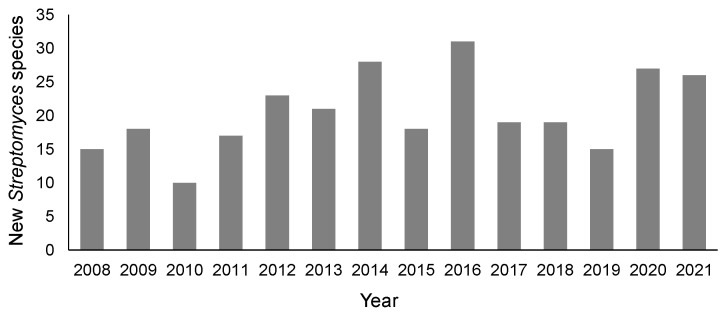
Annual numbers of newly proposed *Streptomyces* species with validly published names. The numbers are based on lists that are annually published in the SAJ News of *Actinomycetologica* (https://www.actino.jp/journal/index.html, accessed on 20 October 2022) No. 1 as the List of new scientific names and nomenclatural change in the phylum *Actinobacteria* (*Actinomycetota* from 2022) validly published in each year.

The genus *Streptomyces* is the largest taxa among prokaryotes. As of March 2005, 487 species and 39 subspecies with validly published names had been approved. A total of 533 species are described in *Bergey’s Manual of Systematic Bacteriology* (Second Edition, Volume Five, The *Actinobacteria*, Part B) issued in 2012. The number increased year by year because several dozen species were newly proposed every year (Figure 1). According to the List of Prokaryotic names with Standing in Nomenclature (LPSN), the number of species with validly published and correct names was 699 as of January 2023. If this number is not limited to those with a correct name, but also includes synonyms, the number is 846. *Streptomyces* members are well known and expected as a rich source for pharmacologically useful bioactive compounds due to their production of complex and diverse secondary metabolites. Approximately two-thirds of all known antibiotics are produced by actinomycetes, mainly by *Streptomyces* members [1]. *Streptomyces* strains have been extensively isolated and screened to search for novel bioactive substances because they are a promising source of commercially and medically important secondary metabolites. Consequently, this genus includes a large number of species, which makes taxonomic characterization and identification difficult.

Early classification of *Streptomyces* species mainly relied on morphological observations [2,3,4]. Morphological characteristics such as spore color, spore chain morphology, melanoid pigment production, spore wall ornamentation and the utilization pattern of nine different sugars as a carbon source in physiological tests were recognized as constant and reliable features in classifications. The International *Streptomyces* Project (ISP) published reliable descriptions of type strains of 458 *Streptomyces* species based on the standard criteria to determine species [5,6,7,8]. Later, other basic phenotypic markers such as physiological and biochemical properties, chemotaxonomy and DNA–DNA hybridization (DDH) of total chromosomal DNA were employed for classification. In particular, DNA–DNA relatedness (DNA–DNA reassociation) in DDH is the gold standard for species identification because a clear and objective numerical threshold, 70%, is given and widely recognized as the species boundary [9,10]. In the 1980s, 16S rRNA gene sequence analysis was applied for bacteria systematics. Phylogenetic analysis based on 16S rRNA gene sequences and determination of the sequence similarities are nowadays carried out as the first step in identification. These methods do not have sufficient resolution to delimit *Streptomyces* species. In actinomycetes, strains showing less than 99% similarities in their 16S rRNA gene sequences can be considered to be different species [11]. In contrast, even if 16S rRNA gene sequence similarity exceeds 99% between two strains, it is unclear whether they belong to the same species or not. It is often reported that some species share a high 16S rRNA gene sequence similarity, such as 100% and 99.9%, even though they are clearly separated as different species by DNA–DNA relatedness [12]. Hence, it is hard to differentiate two species by 16S rRNA gene sequences alone in many cases. In the current taxonomic criteria, we need to examine DNA–DNA relatedness by DDH for type strains of closely related species showing high 16S rRNA gene sequence similarities. In the genus *Streptomyces*, many species are included as members showing >99% similarities. As DDH is labor-intensive and needs to be repeated several times due to its low reproducibility, it is not practical to conduct using many closely related reference strains. This makes the classification more difficult.

There were (and may still be) many species and subspecies whose taxonomic positions needed to be re-evaluated in the genus *Streptomyces*. For example, Tamura et al. revealed that *S. caeruleus* belongs not to the genus *Streptomyces*, but to the genus *Actinoalloteichus*, and reclassified *S. caeruleus* to *A. cyanogriseus* based on the data of phylogenetic analysis of 16S rRNA gene sequences, diagnostic cell-wall diamino acid and DDH in 2008 [13]. In 2010, Kumar and Goodfellow revealed that *S. hygroscopicus* subsp. *angustmyceticus* and *S. hygroscopicus* subsp. *decoyicus* are not *S. hygroscopicus*, but independent species, by analyses of 16S rRNA gene sequences and phenotypic properties, and then reclassified these two subspecies to *S. angustmyceticus* and *S. decoyicus*, respectively [14]. Kim et al. revealed that *S. fimicarius* and *S. setonii* belong to the same species based on 16S rRNA gene and *gyrB* sequences, and reclassified *S. fimicarius* as a synonym of *S. setonii* according to the rules on nomenclature in 2012 [15]. *S. caeruleus*, *S. hygroscopicus* subsp. *angustmyceticus*, *S. hygroscopicus* subsp. *decoyicus* and *S. fimicarius* were published in 1958, 1956, 1959 and 1948, respectively, when molecular approaches such as 16S rRNA gene sequence analysis had not been developed. Similarly, taxonomists often notice the presence of many species that need to be reclassified based on the current criteria in classifications when they review taxonomic positions of species proposed in old days by molecular analysis. However, since classifications of *Streptomyces* members are not easy as stated above, review and reclassification of such species have not gone well until recently. To enable such reclassifications, advances in molecular taxonomy were demanded [16,17].

Very recently, whole genome sequences (WGSs) of many type strains became available due to cost-effective high-throughput whole genome-sequencing platforms and massive whole genome-sequencing projects [18]. Published WGSs of *Streptomyces* strains remarkably increased in numbers from 2014 [19]. WGSs are useful and the ultimate information for taxonomy. They can be used for determination of the overall genome relatedness index (OGRI) [20] and phylogenomic analysis. Despite the increasing number, published WGSs were unfortunately not immediately, effectively and extensively used to reclassify known *Streptomyces* species. However, reports on reclassifications of *Streptomyces* strains increased very recently, and they often include significant insights. This review summarizes and introduces the progress in this decade to update our understanding of the taxonomy of *Streptomyces* members.

## 2. Developments of Molecular Identification Methods

Members of the genus *Streptomyces* had been classified mainly based on phenotypes such as morphologies of colonies and spores, and physiological characteristics in early days. Currently, taxonomy of *Streptomyces* members relies on polyphasic combinations of phenotypic, chemotaxonomic and genotypic characteristics. In the 1980s, 16S rRNA gene sequence analysis became a standard method in bacteria systematics. In this analysis, 16S rRNA genes are amplified by PCR, and subsequently the amplicons are sequenced by the Sanger method. The retrieved gene sequence is analyzed by BLAST to search for closely related species. The sequence is also used to reconstruct a phylogenetic tree to clarify the phylogenetic position among close neighbors. In 2012, Labeda et al. determined the almost complete 16S rRNA gene sequences of a wide variety of *Streptomyces* members, including almost all described species, and reconstructed a comprehensive phylogenetic tree to clarify relationships between type strains of closely related *Streptomyces* species, demonstrate the diversity of the species and allow selection of representative strains for further studies in a collaborative study [21]. However, the resolution of 16S rRNA gene sequence analysis is known to be too low to identify strains at the species level. The 16S rRNA gene sequence similarity value of 99% is a cut-off to classify as a novel species when compared with the phylogenetically closest neighbors with validly published names [11]. However, there is no guarantee for strains showing more than 99% to belong the same species, although those showing less than 99% in the similarity on 16S rRNA gene sequences can be considered as different species [22].

As alternatives to 16S rRNA gene sequence analysis, sequence analyses of protein-coding housekeeping genes were developed for higher resolutions. Kasai et al. employed *gyrB* gene sequences to study intrageneric relationships among *Micromonospora* species. A *gyrB* segment about 1.2 kb long was amplified by PCR using universal primers, and the sequence was determined. It is reported that a genetic distance of about 0.0014, roughly equivalent to 98.5% similarity, in a *gyrB* gene sequence corresponded to 70% DNA–DNA relatedness. Furthermore, strains with approximately 97% or higher *gyrB* sequence similarity and similar phenotypes in a clade exhibited DNA–DNA relatedness of >65%, an acceptable value for proposal of a single species [23]. Hatano et al. used *gyrB* gene sequences in a taxonomic re-evaluation of whorl-forming *Streptomyces* species. They amplified 1.2 kb of *gyrB* using primers, different from those of Kasai et al., by PCR and the amplicons were sequenced. Consequently, a correlation between the sequence similarities of the *gyrB* gene and DNA–DNA relatedness by DDH was also observed in these members. They stated that strains with 95.5–96.5% *gyrB* sequence similarity and similar phenotypes in a clade exhibited DNA–DNA relatedness of 59–75%.

Multilocus sequence analysis (MLSA) has been demonstrated as a valuable tool for assessing species assignments in prokaryote taxonomy. The resolution is significantly improved by using multiple gene sequences compared to those based on a single gene sequence [24]. Guo, Rong, Huang et al. reported MLSA for the genus *Streptomyces*. They amplified five housekeeping genes, *atpD*, *gyrB*, *recA*, *rpoB* and *trpB*, by PCR, sequenced the amplicons and used DNA sequences of 496 bp (or 495 bp), 423 bp (or 422 bp), 504 bp, 540 bp and 573 bp (or 567 bp) from these five genes, respectively [25,26]. Phylogenetic trees using the concatenated five gene sequences of 2526 bp (or 2534 bp) showed robust phylogenetic relationships. The evolution distance of 0.007 in this MLSA corresponded to DNA–DNA relatedness of 70% [26]. Phylogenetic analysis based on MLSA is very useful to find closely related reference strains based on their phylogenetic relationships. DNA–DNA relatedness by DDH is the gold standard for species identification. There are many cases that DDH needs to be conducted, not only for the closest reference, but also for many references, because 16S rRNA gene similarities are higher among closely related species in the genus *Streptomyces*. However, since DDH experiments with many combinations are not practical. MLSA is also useful to determine the most closely related references that need to be compared in detail. The potential of MLSA in bacterial species definition was strongly endorsed by the ad hoc committee of the International Committee on Systematics of Prokaryotes [17], given that whole-genome sequencing was not accessible to most microbial taxonomists at that time. MLSA has been demonstrated as a valuable tool for assessing species assignments in the genus *Streptomyces* [25,26,27,28,29,30]. It can be utilized as a replacement for DDH [24].

The availability of WGSs allows us to choose many housekeeping genes for MLSA. Nowadays, MLSA using many housekeeping gene sequences can be carried out because lots of Web tools are available [31,32,33,34,35] Many of them automatically extract housekeeping gene sequences from WGSs and reconstruct the phylogenetic trees based on the concatenated sequences. MLSA is more phylogenetically sensible and can be a good alternative to check conclusions derived from the overall genome relatedness index (OGRI) [20].

DNA–DNA relatedness had been determined by DDH experiments before. The microplate hybridization method developed by Ezaki et al. [36,37] was often applied to determine DNA–DNA relatedness. However, DDH experiments have a problem in their reproducibility. Nowadays, whole genome sequencing is affordable in general laboratories. WGSs of many type strains are accessible due to a massive whole genome sequencing project focusing on type strains of bacterial species [18]. Furthermore, since in order to propose new species in *Int. J. Syst. Evol. Microbiol.*, researchers have to sequence the whole genome of its type strain and deposit it to GenBank/ENA/DDBJ for publication, WGSs of recently proposed new species become available immediately. DDH experiments can be easily replaced by determination of OGRI such as average nucleotide identity (ANI) and DNA–DNA relatedness based on digital DDH (dDDH). As these methods are not experiments in a wet lab, but in silico analyses, the results are reproducible and objective. It is also feasible to determine these values easily even for many strain pairs by using a personal computer. ANI represents a mean of identity values between multiple sets of orthologous regions shared by two genomes [38]. Based on comparative studies between ANI and DNA–DNA relatedness based on DDH, ANI values equivalent to 70% DNA–DNA relatedness are 95–96% [39,40]. Software such as JSpecies, ANI calculator and OrthoANI are available for ANI [40,41]. On the other hand, the Genome-to-Genome Distance Calculator (GGDC) is a Web service used to calculate DNA–DNA relatedness, called a DDH estimate, by dDDH [42] and can be used instead of DDH experiments. ANI and DDH estimates by the GGDC are representative OGRI to indicate how similar two genome sequences are [43]. TYGS, a Type (Strain) Genome Server, was developed as a user-friendly high-throughput Web server for genome-based prokaryote taxonomy and is available on the Web. When users submit a job by uploading a WGS(s) as a query (queries) on the site, TYGS provides many data, such as results on the identification, a phylogenetic tree based on 16S rRNA gene sequences, a tree based on WGSs and pairwise comparisons to close known species. Although this analysis is state-of-the-art [44], users need to note that WGSs of some of the closely related species have not been made available or registered in the dataset.

G + C content of genomic DNA is historically essential in bacterial taxonomy and was determined by experiments using HPLC. Nowadays, the precise content can be calculated from WGS(s).

## 3. Materials and Methods

Reclassified *Streptomyces* strains stated in this review were searched from the List of new scientific names and nomenclatural change in the phylum *Actinobacteria* (*Actinomycetota* from 2022) validly published in each year of the annually published SAJ News of *Actinomycetologica* No. 1 (https://www.actino.jp/journal/index.html). Phylogenetic trees were reconstructed by ClustalX 2.1. Representative species of each *Streptomyces* group listed in the Table 11 titled ‘List of *Streptomyces* species (including *Kitasatospora* and *Streptacidiphilus* species) arranged according to the grouping given in Figure 21’ in the Genus *Streptomyces* of *Bergey’s Manual of Systematics of Archaea and Bacteria* were included in the figure of Section 4.1 to show the comprehensive phylogenetic positions of *Streptomyces* members. Subspecies with validly published names in the genus *Streptomyces* were searched using LPSN to reconstruct a phylogenetic tree shown in Section 4.2.

## 4. Recent Reclassification of Members in the Genus *Streptomyces*

Members in the genus *Streptomyces* reclassified in the past decade are grouped into three categories: (i) transferred from the genus *Streptomyces* to the other genera, (ii) subspecies, and (iii) unified as synonyms of other species. They are overviewed in the following sections with list tables (Table 1, Table 2 and Table 3, respectively).

### 4.1. Transfer to Other Genera

In 2017, Labeda et al. reconstructed a large phylogenetic tree of members in the family *Streptomycetaceae* based on MLSA using *atpD*, *gyrB*, *recA*, *rpoB* and *trpB* gene sequences. The tree includes type strains of approximately 590 *Streptomyces* species and subspecies, six *Kitasatospora* species and four *Streptacidiphilus* species with validly published names. This analysis phylogenetically clarified the distinctiveness of the genera *Kitasatospora* and *Streptacidiphilus*, which are closely related to the genus *Streptomyces*. Furthermore, nine *Streptomyces* species were transferred into the genus *Kitasatospora*: *S. aburaviensis*, *S. albolongus*, *S. aureofaciens*, *S. avellaneus*, *S. cinereorectus*, *S. herbaricolor*, *S. misakiensis*, *S. psammoticus* and *S. purpeofuscus* were reclassified to *K. aburaviensis* comb. nov., *K. albolonga* comb. nov., *K. aureofaciens* comb. nov., *K. aureofaciens*, *K. cinereorecta* comb. nov., *K. herbaricolor* comb. nov., *K. misakiensis* comb. nov., *K. psammotica* comb. nov. and *K. purpeofusca* comb. nov., respectively, as they are now included in a clade specific to members in the genus *Kitasatospora* (Table 1). Since *S. aureofaciens* and *S. avellaneus* were not discriminated as two independent species, the same species name, *K. aureofaciens*, was given to them [45].

In 2018, Nouioui et al. reported WGS-based reclassification of the phylum *Actinobacteria*. In this report, already published draft genome sequences of a large collection of actinobacterial type strains were used to reconstruct phylogenomic trees. Mainly based on the phylogenomic relationships, many members of not only the genus *Streptomyces*, but also other genera, were reclassified in this study. As transfers from the genus *Streptomyces* to other genera, *S. indigoferus* and *S. xanthocidicus* were transferred to the genus *Kitasatospora*, and, consequently, new combinations *Kitasatospora indigofera* comb. nov. and *Kitasatospora xanthocidica* comb. nov. were proposed, respectively. *S. scabrisporus* and *S. aomiensis* were transferred to new genera, *Embleya* gen. nov. and *Yinghuangia* gen. nov., respectively [46]. Until then, the family *Streptomycetaceae* was composed of only four genera: *Allostreptomyces*, *Kitasatospora*, *Streptacidiphilus* and *Streptmyces*. By establishing the new genera *Embleya* and *Yinghuangia*, this family included six genera. This study mainly relied on massive phylogenomic analyses. In contrast, characteristics specific to each established taxon appear to be less focused on. Whole genome sequence-based reclassification may be a recent trend of actinomycetal systematics.

In the next year, Nouioui et al. transferred *S. griseoplanus* to the genus *Streptacidiphilus* because the authors noticed that *S. griseoplanus* is not *Streptomyces*, but belongs to the genus *Streptacidiphilus* by phylogenetic analysis based on 16S rRNA gene sequences when proposing a novel *Streptacidiphilus* species, *Streptacidiphilus bronchialis* sp. nov. [47]. Komaki et al. transferred *S. catbensis* and *S. seranimatus* to the genus *Yinghuangia* as *Yinghuangia catbensis* comb. nov. and *Yinghuangia seranimata* comb. nov., respectively [48], by phylogenetic analysis of 16S rRNA gene sequences and morphological, physiological, biochemical and chemotaxonomic data, since these two species had been left in the genus *Streptomyces* in the report by Nouioui et al. [46].

In 2020, Komaki et al. proposed *Embleya hyalina* sp. nov. by transfer of “*S. hyalinum*”, a *Streptomyces* species without a validly published name, to the genus *Embleya.* This is the second species in the genus *Embleya*. In this study, the authors sequenced the whole genome of “*S. hyalinum*” NBRC 13850^T^ and examined its characteristics, such as chemotaxonomic, biochemical and physiological features, in detail [49]. *Embleya* sp. NF3 is a steffimycin producer [50] and its whole genome sequence is published in GenBank/ENA/DDBJ. Although this strain is registered as *E. scabrispora* NF3 in GenBank/ENA/DDBJ, it is not *E. scabrispora* because the DNA–DNA relatedness between *Embleya* sp. NF3 and *E. scabrispora* DSM 41855^T^ is 31%, much lower than the threshold for species delineation. As *Embleya* sp. NF3 is also not *E. hyalina*, since its DNA–DNA relatedness to *E. hyalina* NBRC 13850^T^ is 54% (unpublished), *Embleya* sp. NF3 should be proposed as a new independent species, although a species description based on specific characteristics is required. Reports on bioactive substances from this genus are increasing in recent years [51,52]. Thus, members of the genus *Embleya* will attract attention as a rich source for new bioactive secondary metabolites, and, consequently, more new species will be discovered.

In 2021, Teo et al. transferred *S. caeruleus* to the genus *Actinoalloteichus* and then named *Actinoalloteichus caeruleus* comb. nov. in a study proposing novel genera by phylogenomic analyses [53]. *Actinoalloteichus cyanogriseus*, proposed by Tamura et al. in 2000 [54], is a synonym of *A. caeruleus.* Volpiano et al. reclassified *S. thermoautotrophicus* to a novel genus, the genus *Carbonactinospora* gen. nov., and named it *Carbonactinospora thermoautotrophica* comb. nov. In this report, a novel family, the family *Carbonactinosporaceae* fam. nov., was also proposed, including the genus and species derived from *S. thermoautotrophicus* [55].

In 2022, Madhaiyan et al. transferred a group of 12 *Streptomyces* species (*S. yeochonensis*, *S. acididurans*, *S. alni*, *S. bryophytorum*, *S. epipremni*, *S. glauciniger*, *S. guanduensis*, *S. oryziradicis*, *S. paucisporeus*, *S. rubidus*, “*S. soli*”, *S. yanglinensis*), “*S. gilvigriseus*”, “*S. cattleya*” and two species (*S. vitaminophilus* and *S. tyrosinilyticus*) to newly proposed genera *Actinacidiphila* gen. nov., *Mangrovactinospora* gen. nov., *Streptantibioticus* gen. nov. and *Wenjunlia* gen. nov., respectively, based on phylogenomic analyses coupled with phenotypic comparisons, citing previous reports. They also proposed two other new genera, *Peterkaempfera* gen. nov. and *Phaeacidiphilus* gen. nov., through the reclassification of three *Streptacidiphilus* species. Consequently, the family *Streptomycetaceae* includes 12 genera at present [56]. High resolutions of whole genome sequence-based classifications enable us to subdivide the genus *Streptomyces* with closely related taxa although these taxa are large and complicated.

**Table 1:** *Streptomyces* species transferred to other genera.

Species	Reclassified to	Reference
*S.* *aburaviensis*	*Kitasatospora aburaviensis* comb. nov.	Labeda et al., 2017 [45]
*S. albolongus*	*Kitasatospora albolonga* comb. nov.	
*S. aureofaciens*	*Kitasatospora aureofaciens* comb. nov.	
*S. avellaneus*	*Kitasatospora aureofaciens*	
*S. cinereorectus*	*Kitasatospora cinereorecta* comb. nov.	
*S. herbaricolor*	*Kitasatospora herbaricolor* comb. nov.	
*S. misakiensis*	*Kitasatospora misakiensis* comb. nov.	
*S. psammoticus*	*Kitasatospora psammotica* comb. nov.	
*S. purpeofuscus*	*Kitasatospora purpeofusca* comb. nov.	
*S. indigoferus*	*Kitasatospora indigofera* comb. nov.	Nouioui et al., 2018 [46]
*S. xanthocidicus*	*Kitasatospora xanthocidica* comb. nov.	
*S. scabrisporus*	*Embleya scabrispora* gen. nov., comb. nov.	
*S. aomiensis*	*Yinghuangia aomiensis* gen. nov., comb. nov.	
*S. griseoplanus*	*Streptacidiphilus griseoplanus* comb. nov.	Nouioui et al., 2019 [57]
*S. catbensis*	*Yinghuangia catbensis* comb. nov.	Komaki et al., 2019 [48]
*S. seranimatus*	*Yinghuangia seranimata* comb. nov.	
“*S. hyalinum*”	*Embleya hyalina* sp. nov.	Komaki et al., 2020 [49]
*S. caeruleus*	*Actinoalloteichus caeruleus* comb. nov.	Teo et al., 2021 [53]
*S. thermoautotrophicus*	*Carbonactinospora thermoautotrophica* gen. nov., comb. nov.	Volpiano et al., 2021 [55]
*S. yeochonensis*	*Actinacidiphila yeochonensis*, gen. nov., comb. nov.	Madhaiyan et al. 2022 [56]
*S. acididurans*	*Actinacidiphila acididurans* comb. nov.	
*S. alni*	*Actinacidiphila alni* comb. nov.	
*S. bryophytorum*	*Actinacidiphila bryophytorum* comb. nov.	
*S. epipremni*	*Actinacidiphila epipremni* comb. nov.	
*S. glauciniger*	*Actinacidiphila glaucinigra* comb. nov.	
*S. guanduensis*	*Actinacidiphila guanduensis* comb. nov.	
*S. oryziradicis*	*Actinacidiphila oryziradicis* comb. nov.	
*S. paucisporeus*	*Actinacidiphila paucisporea* comb. nov.	
*S. rubidus*	*Actinacidiphila rubida* comb. nov.	
“*S. soli*”	*Actinacidiphila soli* sp. nov.	
*S. yanglinensis*	*Actinacidiphila yanglinensis* comb. nov.	
“*S. gilvigriseus*”	*Mangrovactinospora gilvigrisea* gen. nov., sp. nov.	
“*S. cattleya*”	*Streptantibioticus cattleyicolor* gen. nov., sp. nov.	
*S. vitaminophilus*	*Wenjunlia vitaminophila* gen. nov., comb. nov.	
*S. tyrosinilyticus*	*Wenjunlia tyrosinilytica* comb. nov.	

**Figure 2:**
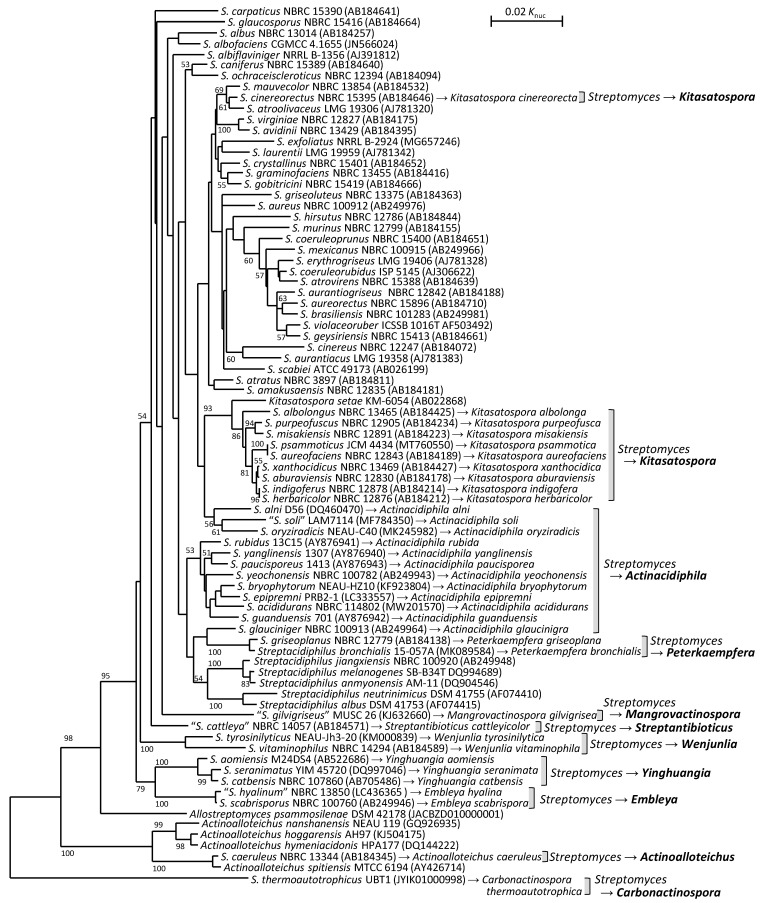
Phylogenetic tree of type strains of *Streptomyces* species transferred to other genera based on 16S rRNA gene sequences. In addition to the members in Table 1, type strains of 35 *Streptomyces* species, *Kitasatospora setae*, four *Streptachidiphilus* species, *Allostreptomyces psammosilienae* and four *Actinoalloteichus* species are included as references. Numbers on branches indicate the percentage bootstrap values of 1000 replicates; only values >50% are indicated.

A phylogenetic tree based on 16S rRNA gene sequences was reconstructed to overview the phylogenetic positions of these *Streptomyces* species transferred to other genera (Figure 2). This tree includes thirteen genera. *Actinoalloteichus* is a genus of the family *Pseudonocardiaceae*, whereas the remaining twelve are those of the family *Streptomycetaceae*. Members of the genus *Actinoalloteichus* formed an independent clade, which is supported by a bootstrap value of 100%. Eleven genera of the family *Streptomycetaceae* formed a large clade with a 99% bootstrap value, but *Carbonactinospora thermoautotrophica* was unexpectedly not included in it and, rather, located at a position like an outgroup, although *C. thermoautotrophica* has been reported as a member of the family *Streptomycetaceae*. Among ten species transferred to the genus *Kitasatospora*, nine formed a clade with *Kitasatospora setae*, the type species of this genus, but *Kitasatospora cinereorecta* was not included in this clade. The taxonomic positions of *C. thermoautotrophica* and *K. cinereorecta* may need to be re-evaluated in further studies.

### 4.2. Reclassification of Subspecies

**Figure 3:**
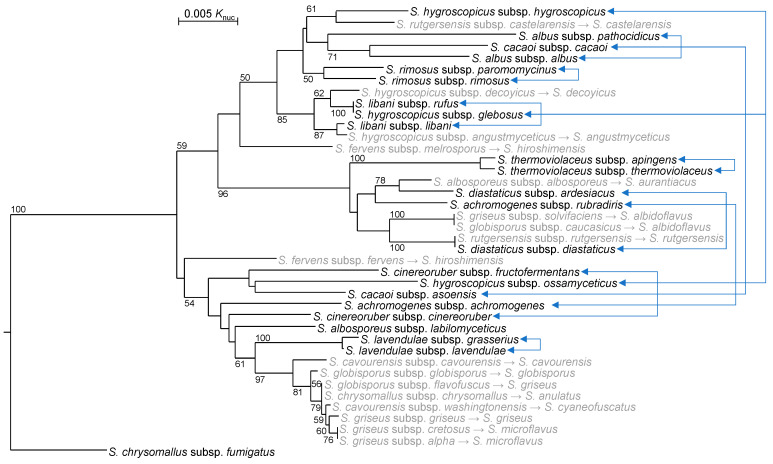
Phylogenetic tree of all *Streptomyces* subspecies with validly published names based on 16S rRNA gene sequences. Subspecies indicated in gray were reclassified to species before 2011, as shown after each gray arrow. Twenty-three subspecies present in 2012 are indicated in black. Subspecies pairs (or trios) within the same species are connected by light blue lines with double arrows in this figure. Numbers on branches indicate the percentage bootstrap values of 1000 replicates; only values >50% are indicated. *Kitasatospora setae* NBRC 14216^T^ (AB184576.2) is used for an outgroup (not shown) to indicate the root of this tree.

The genus *Streptomyces* once included at maximum 39 subspecies. Among them 17 subspecies were reclassified to independent new species or synonyms of already recognized species before 2011 (gray in Figure 3). However, many subspecies remained unreviewed before this decade, although seven subspecies pairs (or trios) were not phylogenetically close (as connected by light blue lines with double arrows in Figure 3).

**Figure 4:**
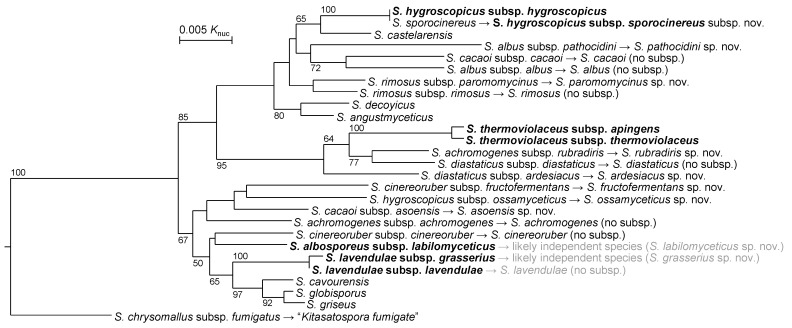
Phylogenetic tree of the members in Figure 3 with updated names based on 16S rRNA gene sequences. As members reclassified as synonyms and a new subspecies (*S. hygroscopicus* subsp. *sporocinereus*) were removed and added, respectively, this tree includes only the present type strains. The genus *Streptomyces* still includes seven subspecies at present, shown as boldfaced. Since *S. albosporeus* subsp. *labilomyceticus* and *S. lavendulae* subsp. *grasserius* are likely two novel independent species, *Streptomyces labilomyceticus* sp. nov. and *Streptomyces grasserius* sp. nov., respectively, as shown in gray [47], there are indeed only four subspecies in this genus. Type strains of *S. albidoflavus*, *S. anulatus*, *S. aurantiacus*, *S. cyaneofuscatus*, *S. hiroshimensis*, *S. microflavus*, *S. nigrescens* and *S. platensis* are not included in the same manner as Figure 3 to highlight the results by current reclassifications. The other footnotes, such as descriptions on the numbers on branches and the outgroup, are the same as those in Figure 3.

In 2014, Labeda et al. elevated *S. albus* subsp. *pathocidicus* to *Streptomyces pathocidicus* sp. nov. as an independent species (Table 2). They used MLSA for taxonomic evaluation of *Streptomyces albus* and related taxa. *S. albus* subsp. *pathocidicus* and *S. albus* subsp. *albus* were phylogenetically distant and concluded to be discriminable as different species [28]. Three years later, Labeda et al. expanded their MLSA-based classification from a specific group to the entirety of the genus *Streptomyces*, including other genera such as *Kitasatospora* and *Streptacidiphilus* in the family *Streptomycetaceae*. In this report, *S. chrysomallus* subsp. *fumigatus* was transferred to the genus *Kitasatospora* and elevated as an independent species, “*Kitasatospora fumigate*” comb. nov., but the name is not validly approved yet [45]. From 2013, available whole genome sequences of *Streptomyces* strains increased in number [19], but whole genome sequences of many type strains in this genus remain unread and/or unpublished yet. Komaki et al. sequenced whole genomes of nine subspecies type strains whose WGSs had not been sequenced or published and reviewed their taxonomic positions. Consequently, seven subspecies, namely *S. rimosus* subsp. *paromomycinus*, *S. diastaticus* subsp. *ardesiacus*, *S. achromogenes* subsp. *rubradiris*, *S. albosporeus* subsp. *labilomyceticus*, *S. cacaoi* subsp. *asoensis*, *S. cinereoruber* subsp. *fructofermentans* and *S. hygroscopicus* subsp. *ossamyceticus* were reclassified to independent and new species because they did not belong to other species [47,58,59]. *S. rutgersensis* subsp. *rutgersensis*, *S. hygroscopicus* subsp. *glebosus* and *S. libani* subsp. *rufus*, and *S. libani* subsp. *libani* were revealed to be later heterotypic synonyms of known species with validly published names: *S. diastaticus*, *S. platensis*, and *S. nigrescens*, respectively [59,60]. Consequently, there are only five subspecies in the genus *Streptomyces*. However, MLSA suggested that *S. albosporeus* subsp. *labilomyceticus* and *S. lavendulae* subsp. *grasserius* should be reclassified to independent and new species, as shown in gray in Figure 4, after sequencing their whole genomes. To propose a new species, the whole genome sequence of the type strain needs to be published. In practice, only *S. thermoviolaceus* comprised definite subspecies, *S. thermoviolaceus* subsp. *thermoviolaceus* and *S. thermoviolaceus* subsp. *apingens*, as of the end of 2021 [47]. 

Subsequently, Komaki et al. reviewed the taxonomic positions of *S. sporocinereus*, which was a later heterotypic synonym of *S. hygroscopicus*, in comparison with strains such as *Streptomyces* sp. N11-34, *S. hygroscopicus* TP-A0867 and *S. hygroscopicus* NBRC 16556. Consequently, strains in *S. hygroscopicus* were divided into two independent subspecies: *S. hygroscopicus* subsp. *hygroscopicus* and *Streptomyces hygroscopicus* subsp. *sporocinereus* subsp. nov. *S. hygroscopicus* subsp. *hygroscopicus* includes the type strain of *S. endus* as a later heterotypic synonym. *S. hygroscopicus* subsp. *sporocinereus* includes the type strain of *S. sporocinereus*, *S. demainii* as a later heterotypic synonym, and three strains that they examined [61]. In actuality, this is the second subspecies pair of the genus *Streptomyces*. Only subspecies within *S. hygroscopicus* and *S. thermoviolaceus* have been phylogenetically confirmed at present, as shown in Figure 4.

**Table 2:** Reclassification of subspecies in the genus *Streptomyces*.

Taxa	Reclassified to	Reference
*S. albus* subsp. *pathocidicus*	*S. pathocidicus* sp. nov.	Labeda et al., 2014 [28]
*S. chrysomallus* subsp. *fumigatus*	“*Kitasatospora fumigate*” comb. nov.	Labeda et al., 2017 [45]
*S. rimosus* subsp. *paromomycinus*	*S. paromomycinus* sp. nov.	Komaki et al., 2019 [58]
*S. diastaticus* subsp. *ardesiacus*	*S. ardesiacus* sp. nov	Komaki et al., 2020 [59]
*S. achromogenes* subsp. *rubradiris*	*S. rubradiris* sp. nov.	Komaki et al., 2021 [47]
*S. albosporeus* subsp. *labilomyceticus*	*S. labilomyceticus* sp. nov.	
*S. cacaoi* subsp. *asoensis*	*S. asoensis* sp. nov.	
*S. cinereoruber* subsp. *fructofermentans*	*S. fructofermentans* sp. nov.	
*S. hygroscopicus* subsp. *ossamyceticus*	*S. ossamyceticus* sp. nov.	
*S. rutgersensis* subsp. *rutgersensis*	synonym of *S. diastaticus*	Komaki et al., 2020 [59]
*S. hygroscopicus* subsp. *glebosus*	synonym of *S. platensis*	Komaki et al., 2020 [60]
*S. libani* subsp. *rufus*	synonym of *S. platensis*	
*S. libani* subsp. *libani*	synonym of *S. nigrescens*	
*S. sporocinereus*	*S. hygroscopicus* subsp. *sporocinereus* subsp. nov.	Komaki et al., 2022 [61]

### 4.3. Synonym

Overclassification is a problem in the taxonomy of *Streptomyces*. Most species with validly published names were proposed before molecular-based methods are employed. In 2012, Labeda et al. reported phylogenetic relationships among type strains of almost all *Streptomyces* species. In this report, many synonymies between or among different species were suggested. However, as stated by the authors, to reclassify them as synonyms, emended descriptions are required after examining DNA–DNA relatedness to ensure them to be the same species. Thus, Labeda et al. did not reclassify them in this report. Rather, this study provided a road map for planning future genome sequencing for type strains whose whole genome sequences were not available yet [21].

Several research groups reclassified distinct species to synonyms in this decade, as listed in Table 3. In 2012, Kim et al. sequenced 16S rRNA genes and *gyrB* of type strains of 22 species that are phylogenetically close to *S. griseus* and re-evaluated their taxonomic positions. Consequently, *S. albovinaceus* and *S. griseinus* were reclassified as synonyms of *S. globisporus*, whereas *S. fimicarius* was reclassified as that of *S. setonii* [15].

In 2014, Labeda et al. investigated taxonomic relationships among *S. albus* and its related species [28], which showed high 16S rRNA gene sequence similarities in their previous report [21], by MLSA. Consequently, *S. almquistii*, *S. flocculus*, *S. gibsonii* and *S. rangoonensis* were reclassified as synonyms of *S. albus* [28].

In 2017, Labeda et al. evaluated taxonomic relationships of members included in the clade of *S. hirsutus* [21] by MLSA, whole genome sequencing and dDDH. Consequently *S. bambergiensis*, *S. cyanoalbus* and *S. emeiensis* were reclassified to synonyms of *S. prasinus*, *S. hirsutus* and *S. prasinopilosus*, respectively [62]. Komaki et al. sequenced WGSs of type strains of *S. endus*, *S. hygroscopicus* subsp. *hygroscopicus* and *S. sporocinereus* and examined ANIs among the three. As the values exceeded the cut-off for species delineation, *S. endus* and *S. sporocinereus* were reclassified as synonyms of *S. hygroscopicus* subsp. *hygroscopicus* [63]. As the taxonomic position of *S. sporocinereus* was re-evaluated in our further study [61], the synonymy between *S. sporocinereus* and *S. hygroscopicus* subsp. *hygroscopicus* is not included in Table 3. Wink et al. reclassified *S. canchipurensis* as a synonym of *S. muensis*. This synonymy was found in a study to determine the taxonomic position of “*S. caelicus*” DSM 40835^T^, whose species name was not validly published. “*S. caelicus*”, as well as *S. canchipurensis*, was finally reclassified to *S. muensis* [64]. Goodfellow et al. reclassified *S. ghanaensis* as a synonym of *S. viridosporus*. This relationship was revealed in a study characterizing their isolates and proposing *Streptomyces asenjonii* sp. nov. *S. ghanaensis* and *S. viridosporus* were the most closely related species [65]. Idris et al. reclassified *S. melanogenes* to a synonym of *S. noboritoensis*. This study aimed to determine the taxonomic status of their isolate, for which *Streptomyces aridus* sp. nov. is given. *S. melanogenes* and *S. noboritoensis* were closely related species to *S. aridus*. Since the MLSA and phenotypic data showed that *S. melanogenes* and *S. noboritoensis* belong to a single species, this synonymy was also reported [66]. Kämpfer et al. reclassified *S. phaeopurpureus* to a synonym of *S. griseorubiginosus*. As the large number of species with validly published names remains a major practical obstacle in an overall genotypic reclassification of streptomycetes, which can only be resolved by total genome sequence comparisons, the authors sequenced whole genomes of type strains of these two species by focusing on their relationship. MLSA, OGRI analyses and phenotypic comparisons suggested that the two species belong to the same species [67].

Similarly, Kämpfer et al. reclassified *S. ciscaucasicus* to a synonym of *S. canus* in 2018, by conducting whole genome sequencing of the type strains, MLSA, OGRI calculations and phenotypic comparisons [68]. In 2018, Nouioui et al. conducted taxonomic classification using already published WGSs of 1142 strains of the phylum *Actinobacteria* to provide an improved framework for the classification of actinobacteria based on the principles of phylogenetic systematics. In this study, according to high DNA–DNA relatedness by dDDH, ten *Streptomyces* species were reclassified as synonyms of other species, as shown in Table 3 [46]. However, some synonymies had been reported prior to Nouioui et al., as noted in the footnote of Table 3.

In 2019, Cortés-Albayay et al. reclassified *S. helvaticus* to a synonym of *S. chryseus.* because these two species were found to have 100% 16S rRNA gene sequence similarity with DNA–DNA relatedness by dDDH and ANI values of 95.3% and 99.4 %, respectively, while analyzing the phylogenetic position of an isolate, for which a novel species name was given [69].

In 2020, Komaki et al. sequenced WGSs of type strains of eight species in their three studies and reclassified *S. castelarensis* and *S. sporoclivatus*, *S. gougerotii*, and *S. fulvissimus* to synonyms of *S. antimycoticus*, *S. diastaticus* and *S. microflavus*, respectively, based on phylogenetic relationship, OGRI and phenotypic comparisons [59,70,71]. In reference [71], the taxonomic positions of two other WGS-published strains were examined. *S. violaceusniger* NRRL F-8817 was reclassified to *S. antimycoticus. S. violaceusniger* Tü 4113, a spirofungin producer, was revealed to be a new genomospecies distinct from *S. violaceusniger*. Although these two are not type strains, updating their taxonomic positions appropriately is significant to prevent the incorrect identification, which confuse researchers, from spreading. The authors reviewed relationships among ANI, DNA–DNA relatedness by dDDH, and MLSA evolutionary distance. Consequently, clear correlations were observed among them. This is the first report indicating a numerical relationship between MLSA evolutionary distance and OGRI [71]. In references [59,70], the authors analyzed biosynthetic gene clusters (BGCs) of secondary metabolites such as polyketides and nonribosomal peptides in whole genome sequences. Strains reclassified to the same species (synonyms) harbored the same, or quite similar, sets of polyketide synthase (PKS) and nonribosomal peptide synthetase (NRPS) gene clusters in their genomes. In contrast, strains belonging to different species possessed different sets of clusters, even if they showed high 16S rRNA gene sequence similarity, such as 100% and 99.9% [59,70]. These results support the idea reported in the previous study [72]. Saygin et al. reclassified *S. galilaeus* to a synonym of *S. bobili* in a study proposing *Streptomyces cahuitamycinicus* sp. nov. *S. galilaeus* and *S. bobili* were included in phylogenetic analyses of *S. cahuitamycinicus* as related strains, and then their synonymy was noticed. Based on phylogenetic relationships, MLSA evolutionary distance, OGRI determined by dDDH and ANI analysis, the two species were revealed as synonyms [73]. Madhaiyan et al. analyzed taxonomic relationships between the type strains of *Streptomyces* species that showed high 16S rRNA gene sequence similarity. Based on OGRI such as ANI, dDDH value and AAI using published WGS data, *S. griseofuscus*, *S. kasugaensis*, *S. luridiscabiei*, *S. pharetrae* and *S. stelliscabiei* were reclassified to synonyms of *S. murinus*, *S. celluloflavus*, *S. fulvissimus*, *S. glaucescens* and *S. bottropensis*, respectively [74].

In 2021, Komaki et al. investigated taxonomic relationships between *S. cinnamonensis* and *S. virginiae*, among *S. spororaveus*, *S. xanthophaeus* and *S. nojiriensis* and between *S. vinaceus* and *S. cirratus* because 16S rRNA gene sequence similarities in these pairs and trio are 100%, respectively. The authors sequenced the whole genomes of four type strains whose WGSs had not been published, and revealed synonymy between *S. cinnamonensis* and *S. virginiae* based on OGRI such as ANI and dDDH value, reported phenotypes and BGCs of polyketides and nonribosomal peptides in their genomes. In contrast, synonymy was observed neither among *S. spororaveus*, *S. xanthophaeus* and *S. nojiriensis* nor between *S. vinaceus* and *S. cirratus* [75]. Next, Komaki examined taxonomic relationships among *S. costaricanus*, *S. graminearus*, *S. murinus* and *S. phaeogriseichromatogenes*, sharing the same 16S rRNA gene sequences, by MLSA, ANI analysis, dDDH and reported phenotypes. Consequently, *S. costaricanus*, *S. murinus* and *S. phaeogriseichromatogenes* were reclassified to the same species, whereas *S. graminearus* was not. This report included data on PKS and NRPS gene clusters [76]. Komaki also studied taxonomic relationships of phylogenetically complicated members showing high 16S rRNA gene sequence similarities, such as >99.9%, in three clades by MLSA, dDDH and described characteristics. As a result, *S. enissocaesilis*, *S. geysiriensis*, *S. plicatus* and *S. vinaceusdrappus* were reclassified as synonyms of *S. rochei; S. luteus*, *S. flavoviridis*, *S. asterosporus* and *S. viridodiastaticus* as synonyms of *S. mutabilis*, *S. pilosus*, *S. calvus* and *S. albogriseolus*, respectively; and *S. erythrogriseus* and *S. variabilis*, *S. griseorubens* and *S. matensis*, and *S. coelescens*, *S. humiferus* and *S. violaceolatus* as synonyms of *S. griseoincarnatus*, *S. althioticus*, and *S. anthocyanicus*, respectively. This report also introduced many cases showing whether strains with >99.9% 16S rRNA gene sequence similarity were the same species or not, including results previously reported in his research group [12], which may be useful to review the cut-off value of 16S rRNA gene sequence similarity to discriminate species. Li et al. found higher OGRI between type strains of *S. calvus* and *S. aureorectus* than the cut-off of species boundaries during a study of the diversity of endophytic and rhizospheric actinomycetes. Through a comprehensive comparison of phenotypic, chemotaxonomic and physio-biochemical characteristics as well as MLSA and whole genome sequencing, *S. aureorectus* was reclassified to synonym of *S. calvus* [77]. Saygin investigated taxonomic relationships and genome features of members in the *S. aurantiacus* clade based on MLSA, OGRI and phenotypic and biochemical characteristics. Consequently, *S. ederensis* and *S. glomeroaurantiacus* were reclassified to synonyms of *S. umbrinus* and *S. aurantiacus*, respectively [78]. Hu et al. reclassified *S. michiganensis* as a synonym of *S. xanthochromogenes* in a study proposin*g Streptomyces genisteinicus* sp. nov. for an isolate producing genistein. The authors noticed the close relationship between *S. michiganensis* and *S. xanthochromogenes* in phylogenetic trees of the isolates and selected members. The phenotypic, chemotaxonomic and genotypic comparisons revealed the synonymy between the two species [79].

In 2022, Komaki et al. reclassified *S. demainii* and *S. endus* to synonyms of *S. hygroscopicus* subsp. *sporocinereus* and *S. hygroscopicus* subsp. *hygroscopicus*, respectively, according to MLSA evolutionary distance and signature sequences in housekeeping genes when they proposed *Streptomyces hygroscopicus* subsp. *sporocinereus* subsp. nov. [61]. Next, Komaki reclassified *S. anthocyanicus* and *S. tricolor* as synonyms of *S. violaceoruber*, which was revealed by MLSA after resequencing housekeeping genes as well as phenotypic comparison [80]. This report reminds us about the importance to check whether used sequences are correct, even if they are available from public databases.

**Table 3:** Reclassified taxa as later heterotypic synonyms.

Taxa	Reclassified to a Synonym of	Reference
*S. albovinaceus*	*S. globisporus*	Kim et al., 2012 [15]
*S. fimicarius*	*S. setonii*	
*S. griseinus*	*S. globisporus*	
*S. almquistii*	*S. albus*	Labeda et al., 2014 [28]
*S. flocculus*	*S. albus*	
*S. gibsonii*	*S. albus*	
*S. rangoonensis*	*S. albus*	
*S. bambergiensis*	*S. prasinus*	Labeda et al., 2016 [62]
*S. cyanoalbus*	*S. hirsutus*	
*S. emeiensis*	*S. prasinopilosus*	
*S. endus*	*S. hygroscopicus* subsp. *S. hygroscopicus*	Komaki et al., 2017 [63]
*S. canchipurensis*	*S. muensis*	Wink et al., 2017 [64]
*S. ghanaensis*	*S. viridosporus*	Goodfellow et al., 2017 [65]
*S. melanogenes*	*S. noboritoensis*	Idris et al., 2017 [66]
*S. phaeopurpureus*	*S. griseorubiginosus*	Kämpfer et al., 2017 [67]
*S. ciscaucasicus*	*S. canus*	Kämpfer et al., 2018 [68]
*S. citreofluorescens*	*S. anulatus*	Nouioui et al., 2018 [46]
*S. fluorescens*	*S. anulatus*	
*S. avellaneus* ^1^	*S. aureofaciens*	
*S. ciscaucasicus* ^2^	*S. canus*	
*S. phaeopurpureus* ^3^	*S. griseorubiginosus*	
*S. griseolus*	*S. halstedii*	
*S. spheroides*	*S. niveus*	
*S. californicus*	*S. puniceus*	
*S. floridae*	*S. puniceus*	
*S. emeiensis* ^4^	*S. prasinopilosus*	
*S. helvaticus*	*S. chryseus*	Cortés-Albayay et al., 2019 [69]
*S. castelarensis*	*S. antimycoticus*	Komaki et al., 2020 [71]
*S. sporoclivatus*	*S. antimycoticus*	
*S. gougerotii*	*S. diastaticus*	Komaki et al., 2020 [59]
*S. fulvissimus*	*S. microflavus*	Komaki et al., 2020 [70]
*S. galilaeus*	*S. bobili*	Saygin et al., 2020 [73]
*S. griseofuscus*	*S. murinus*	Madhaiyan et al., 2020 [74]
*S. kasugaensis*	*S. celluloflavus*	
*S. luridiscabiei*	*S. fulvissimus*	
*S. pharetrae*	*S. glaucescens*	
*S. stelliscabiei*	*S. bottropensis*	
*S. cinnamonensis*	*S. virginiae*	Komaki et al., 2021 [75]
*S. costaricanus*	*S. murinus*	Komaki, 2021 [76]
*S. phaeogriseichromatogenes*	*S. murinus*	
*S. enissocaesilis*	*S. rochei*	Komaki, 2021 [12]
*S. geysiriensis*	*S. rochei*	
*S. plicatus*	*S. rochei*	
*S. vinaceusdrappus*	*S. rochei*	
*S. luteus*	*S. mutabilis*	
*S. flavoviridis*	*S. pilosus*	
*S. asterosporus*	*S. calvus*	
*S. erythrogriseus*	*S. griseoincarnatus*	
*S. variabilis*	*S. griseoincarnatus*	
*S. griseorubens*	*S. althioticus*	
*S. matensis*	*S. althioticus*	
*S. viridodiastaticus*	*S. albogriseolus*	
*S. coelescens*	*S. anthocyanicus*	
*S. humiferus*	*S. anthocyanicus*	
*S. violaceolatus*	*S. anthocyanicus*	
*S. aureorectus*	*S. calvus*	Li et al., 2021 [77]
*S. ederensis*	*S. umbrinus*	Saygin, 2021 [78]
*S. glomeroaurantiacus*	*S. aurantiacus*	
*S. michiganensis*	*S. xanthochromogenes*	Hu et al., 2021 [79]
*S. endus*	*S. hygroscopicus* subsp. *hygroscopicus*	Komaki et al., 2022 [61]
*S. demainii*	*S. hygroscopicus* subsp. *sporocinereus*	
*S. anthocyanicus*	*S. violaceoruber*	Komaki, 2022 [80]
*S. tricolor*	*S. violaceoruber*	

^1^, The synonymy between *S. avellaneus* and *S. aureofaciens* was reported by Labeda et al. in 2017 and these two were already transferred to the genus *Kitasatospora* as shown in Table 1; ^2^, The synonymy between *S. ciscaucasicus* and *S. canus* was reported by Kämpfer et al. in the same year; ^3^, The synonymy between *S. phaeopurpureus* and *S. griseorubiginosus* was already reported by Kämpfer et al. in 2017; ^4^, The synonymy between *S. emeiensis* and *S. prasinopilosus* was already reported by Labeda et al. in 2016.

**Figure 5:**
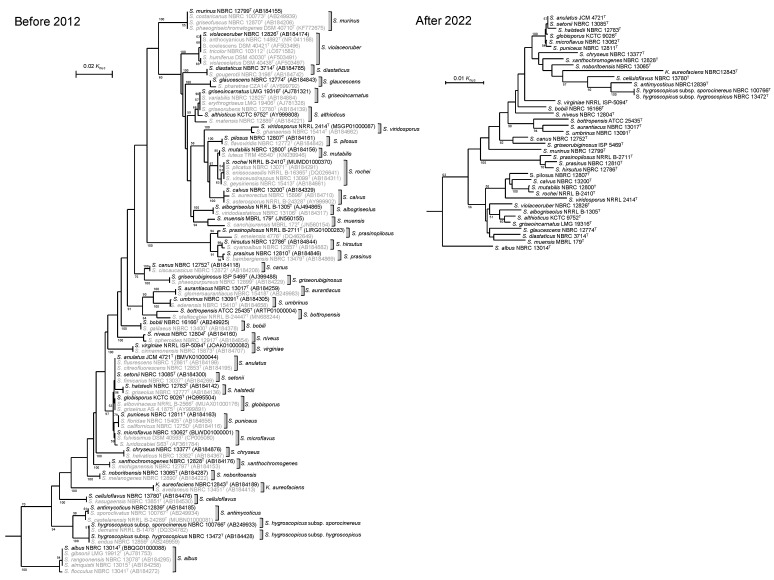
Phylogenetic trees of the type strains of species or subspecies listed in Table 3. Left panel (before 2012): type strains of later heterotypic synonyms are shown in gray. Scientific names with priority (correct names) are in black. Right panel (after 2022): strains identified as later heterotypic synonyms were removed. Numbers on branches indicate the percentage bootstrap values of 1000 replicates; only values >50% are indicated. *Nocardia terpenica* IFM 0706^T^ (JABMCZ010000003) is used for an outgroup (not shown) to indicate the root of this tree.

A phylogenetic tree based on 16S rRNA gene sequences was reconstructed to show relationships between/among species reclassified to the same species as synonyms (Left panel, Figure 5). It is obvious that species with high 16S rRNA gene sequence similarities were reclassified as synonyms because branch lengths are short. Nowadays, members shown in gray can be removed from phylogenetic trees of type strains because they are not type strains with correct names. Consequently, phylogenetic trees will be less complicated than before as a result of the reclassifications stated above (Right panel, Figure 5).

## 5. Further Perspectives

Reviewing classifications based on the latest criteria is essential to prevent taxonomic confusions in systematics. Molecular identification methods using WGSs have improved remarkably in the postgenomic era. This decade was a period that taxonomists applied these methods with initiative and devotedly contributed to *Streptomyces* reclassification, which had not been sufficiently updated despite some molecular studies that suggested its necessity before. Consequently, many species that needed to be re-evaluated are becoming well-organized, as introduced here. In this decade, eight new genera in the family *Streptomycetaceae* were proposed through transfers of *Streptomyces* species. Most subspecies were appropriately reclassified to independent species or synonyms of known species. Identical species with different names were reclassified to synonyms as shown in Table 3 and Figure 5. However, the genus *Streptomyces* may still include species that are unreviewed by molecular data, since this genus is composed of about 700 species and it is not easy to find species with nonupdated names. As 16S rRNA gene sequence analysis may not be sufficient to find synonymous species that have not been reviewed and WGS comparison is not yet feasible for comprehensive analysis, alternative methods are required to accelerate reviewing and updating the classification. Komaki reported a possibility that *trpB* gene sequence similarities can substitute for dDDH [80]. It may be useful to look for species that need to be re-evaluated comprehensively. This is because sequence analysis of a single housekeeping gene is easier and fits comprehensive analysis better than MLSA and OGRI analysis.

*Streptomyces* strains are a rich source for pharmacologically useful bioactive compounds, but their secondary metabolites are not focused on in the taxonomy because it is believed that there is no relationship between taxonomic classification and their products. It is not feasible to explain the relationship because products of a strain often differ when the strain is cultured in different conditions. In contrast, there are many species known to produce specific secondary metabolites. For instance, *S. griseus*, *S. avermitilis* and *S. tsukubensis* are well known as producers of streptomycin, avermectin and tacrolims, respectively. As the other species may produce these compounds, the products cannot be considered as a taxonomic characteristic specific to the species.

**Figure 6:**
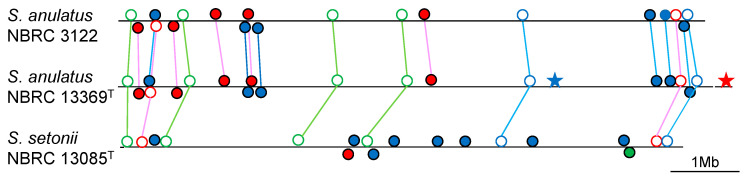
PKS and NRPS gene clusters on linear chromosomes of three strains showing 100% 16S rRNA gene sequence similarity. Red, PKS gene cluster; blue, NRPS gene cluster; green, hybrid PKS/NRPS gene cluster; open circle, conserved between different species; closed circle, specific to each species; star, strain-specific. The same gene clusters are connected by colored lines. All 19 clusters of *S. anulatus* NBRC 3122 are conserved in *S. anulatus* NBRC 13369^T^. Between the two *S. anulatus* strains, only two clusters are specific to strain NBRC 13369^T^. Among the 19 to 21 and 18 clusters of the *S. anulatus* strains and *S. setonii* NBRC 13085^T^, respectively, only eight are conserved between the two species. Between the two species, 11 to 13 are specific to *S. anulatus* whereas 10 are specific to *S. setonii*. Although strain NBRC 3122 is published as *S. griseus* in the NBRC Culture Catalog, it is not *S. griseus*, but *S. anulatus*, because its DNA–DNA relatedness to *S. griseus* DSM 40236^T^, *S. anulatus* NBRC 13369^T^ and *S. setonii* NBRC 13085^T^ is 48.7%, 91.5% and 44.3%, respectively, by dDDH.

When Yamamura et al. proposed a new species, they briefly compared the secondary metabolite-biosynthetic gene clusters (smBGCs) encoded in its whole genome with those of the closest neighbor in their report [81]. To the best of my knowledge, this is the first taxonomical report including data on smBGCs. Subsequently, our research group often employed more detailed analysis of smBGCs, focusing on PKS and NRPS gene clusters in taxonomic studies. Consequently, it was revealed that strains within an identical species harbor the same or very similar sets of PKS and NRPS gene clusters. This is suggestive that the strain-diversities of these gene clusters are not so high within a species. In contrast, strains belonging to different species never harbor the same or very similar sets of these gene clusters, even if the strains are phylogenetically close, such as taxonomic neighbors [59,60,61,70,72,75,76,82,83,84]. An example is shown as Figure 6 (unpublished). The conservation rate of PKS and NRPS gene clusters correlates to DNA–DNA relatedness between strains in general [84]. If strains within an identical species were observed to harbor different sets of PKS and NRPS gene clusters, or if strains of different species possess the same or quite similar sets of these gene clusters, we need to doubt classification of these species (Komaki et al., submitted). Similarly to morphological, physiological, biochemical and chemotaxonomic features, smBGCs such as PKS and NRPS gene clusters in genomes may also become a characteristic to classify *Streptomyces* strains at the species level in the near future.

## Abbreviations

AAI, average amino acid identity; ANI, average nucleotide identity; BGC, biosynthetic gene cluster; dDDH, digital DNA–DNA hybridization; DDH, DNA–DNA hybridization; ISP, International *Streptomyces* Project; MLSA, multilocus sequence analysis; NRPS, nonribosomal peptide synthetase; OGRI, overall genome relatedness index; PKS, polyketide synthase; smBGC, secondary metabolite-biosynthetic gene cluster; TYGS, Type (Strain) Genome Server; GGDC, Genome-to-Genome Distance Calculator; WGS, whole genome sequence.
